# Retrospective Analysis of the Development of Human Thyroglobulin during Pregnancy in Patients with Treated Non-Recurrent Differentiated Thyroid Cancer

**DOI:** 10.3390/curroncol29060320

**Published:** 2022-05-31

**Authors:** Justus Baumgarten, Christian Happel, Daniel Groener, Jennifer Staudt, Benjamin Bockisch, Amir Sabet, Frank Grünwald, Thomas Rink

**Affiliations:** 1 Department of Nuclear Medicine, University Hospital, Goethe University, Theodor Stern Kai 7, D-60590 Frankfurt, Germany; justus.baumgarten@kgu.de (J.B.); daniel.groener@kgu.de (D.G.); benjamin.bockisch@kgu.de (B.B.); amir.sabet@kgu.de (A.S.); frank.gruenwald@kgu.de (F.G.); rink@em.uni-frankfurt.de (T.R.); 2 Department of Nuclear Medicine, Medizinisches Versorgungszentrum (MVZ), D-63739 Aschaffenburg, Germany; langer@nukendo.de; 3 Institute for Nuclear Medicine, Nussallee 7, D-63450 Hanau, Germany

**Keywords:** differentiated thyroid carcinoma, thyroidectomy, radioiodine therapy, thyroglobulin, pregnancy

## Abstract

Aim: Therapy success in patients with differentiated thyroid cancer (DTC) after thyroidectomy and radioiodine therapy (RIT) is proven by permanent decrease in human thyroglobulin (hTg) to <1 ng/mL. In this retrospective analysis hTg development before, during and after pregnancy were analyzed. Material and methods: A descriptive analysis of hTg courses in 47 women with 57 pregnancies under levothyroxine substitution was performed after treatment of DTC without evidence of residual or recurrent disease. We compared hTg levels before, during and after pregnancies. A median of four measurements were performed during pregnancy. Results: In five out of the 47 patients at least one hTg increase to ≥1.0 ng/mL occurred during pregnancy (P1: 1.1; P2: 1.75; P3: 1.0; P4: 1.1; P5: 1.07 ng/mL). In another three cases an increase to ≥0.5 ng/mL occurred. After delivery, all patients returned to undetectable hTg levels. Human Tg maxima during pregnancy were significantly elevated according to Friedman´s Chi2 and p Holm–Bonferroni. Conclusion: In women with ablative thyroid therapy after DTC, a temporary elevation in hTg levels during pregnancy may occur. The reason therefore remains unclear and requires further investigation.

## 1. Introduction

Radioiodine therapy (RIT) is a well-established integral part of therapy in patients with benign and malignant thyroid diseases [1,2,3,4,5]. Based on the German Guideline [1], all differentiated thyroid cancers (DTC) except non-metastatic papillary microcarcinoma are usually treated with thyroidectomy and, depending on the tumor stage, at least one RIT [6]. Diagnostic ^131^I scans are performed for ablation control as well as further diagnostic stratification in case of suspicious findings during follow-up [5,7]. The most important components of follow-up routine are sonography of the neck and blood tests. National and international guidelines make different recommendations for post-treatment thyrotropin (TSH) levels. The American Thyroid Association (ATA) recommends TSH suppression for high risk patients; according to their definition those patients with an extensive extrathyroidal growth, distant metastases, an incomplete tumor resection, a very high postoperative human thyroglobulin (hTg), an N1-situation with metastases > 3 cm, or a follicular carcinoma with extensive vascular invasion (>4 foci) [8]. The tumor marker in DTC is human thyroglobulin, a glycoproteine synthesized by both healthy thyroid and DTC cells [9]. With rising TSH levels, the sensitivity for the detection of hTg increases. About 25 % of patients have elevated thyroglobulin antibodies (TgAB) [10], which can interfere with the measurement. This is why TgAB should always be determined in follow-up examinations [1]. A rising TgAB level even without hTg elevation can be indicative of recurrent or persistent disease. Nevertheless, hTg can also be measured in the presence of significantly elevated TgAB [11]. In order to minimize the probability of a measurement error due to an influence of antibodies, a recovery test is carried out regularly. An hTg recovery rate between 70 and 130 percent is considered normal and makes incorrect measurements unlikely [12]. The success of DTC therapy is shown amongst other parameters by persistent decrease in hTg levels to very low or even undetectable values. In most test kits 1 ng/mL is the upper limit. Values exceeding 1 ng/mLare indicative of residual tumor tissue or metastasis. In some highly sensitive kits, even an increase to over 0.5 ng/mL is suspicious for tumor tissue. Metastases or locally recurrent disease usually have a correlate in the ^131^I whole body scan [5]. Follow-up examinations are highly relevant in pregnant women, as studies suggest a higher persistence or recurrence rate during and after pregnancy [13]. In addition, during pregnancy the need for thyroid hormones may rise. Frequent laboratory controls are needed to warrant sufficient hormone supply. In a previously published case study, an increase in hTg level from 0.23 ng/mL to 1.7 ng/mL was observed during pregnancy in a patient with DTC after thyroidectomy and RIT. One month after delivery hTg level fell to the pre-pregnancy level [14]. The aim of this retrospective analysis is to evaluate hTg in patients with DTC before, during and after pregnancy. Therefore, the influence of pregnancy on laboratory follow-up examinations in DTC was analyzed statistically.

## 2. Materials and Methods

Human Tg courses before, during and after the pregnancies of 47 women were evaluated after the approval of the local ethics committee. Pregnancy occurred in all patients after successful RIT confirmed by a negative stimulated hTg determination. Inclusion criteria were a total thyroidectomy with a minimum of one RIT, no evidence of persistent or recurrent disease and at least one pregnancy after the last RIT. All tumor stages except unifocal non-metastatic pT1a were included. 

Thyroglobulin was measured regularly in all patients at least once before (median 13 weeks), during (median 4 measurements per pregnancy) and after (median 15 weeks) pregnancy. To evaluate the influence of pregnancy, hTg, TSH and hTg recovery rate were included in the study. Human Tg levels were measured using the immunoradiometric assay RIASON Tg c.t. (IASON GmbH, Graz-Seiersberg, Austria). Recovery rates were determined using the standard calibration fluid provided with the Tg-assay, which contains hTg at a concentration of 500 ng/mL. This coated tube test utilizes two special monoclonal anti-Tg-antibodies. One is immobilized on the inner tube surface; the other is used as a ^125^I tracer. Both antibodies bind hTg as a sandwich-complex. After washing, the remaining radioactivity in the tubes was quantified. The analytical assay sensitivity was 0.05 ng/mL. TSH was determined using an immunoradiometric assay (Brahms, Henningsdorf, Germany). The analytical assay sensitivity was 0.04 mIU/L with a reference range from 0.3 to 4.0 mIU/L.

Statistical evaluation was performed by a descriptive analysis of the tumor marker hTg over time. Medians, minima and maxima were determined. Ladder plots were used for graphical illustration. To compare hTg during, before and after pregnancy, a non-parametric Friedman test was used. For all statistical analyses a 5 percent level of significance was used. All statistical evaluations were performed with the dedicated statistical software BIAS.

## 3. Results

Forty-seven patients with DTC from two nuclear medicine departments were evaluated in a retrospective analysis. The patient cohort consisted of 45 patients with papillary and two patients with follicular thyroid carcinoma. Eleven patients had initially diagnosed low-risk tumors (pT1/2, N0, M0); the other patients had high-risk tumors (pT3/4, N+ or M+) according to ATA guidelines [8]. Three patients had pulmonary metastases. TNM of one patient remained unknown.

Human Tg in 45 of these patients was below 0.3 ng/mL before conception, which occurred in median 50 months after the last RIT (range 12–190 months). Baseline characteristics are detailed in Table 1. In two women, initial hTg levels of 0.5 and 0.7 ng/mL were detected prior to pregnancy. The first patient had undergone surgery for an advanced papillary thyroid carcinoma (stage pT4 pN1b M1, with pulmonary metastasis). Afterwards, she underwent surgery for local recurrence three times and had four RIT. Follow-up then remained without evidence of further thyroid malignancy. The second woman did not show any hTg > 0.1 ng/mL during and after pregnancy.

All patients had TSH levels according to ATA recommendations at the beginning of the pregnancy. Thirty-six patients had no TSH suppression during pregnancy, ten of them with at least one TSH value exceeding 4.5 mIU/L, which led to a referral to our outpatient clinics. No TSH elevation above the upper limit occurred with a simultaneous hTg elevation. In all patients, T3 and T4 were consistently in a normal range. In 46 patients, TgAB remained negative or only <10 % elevated above the upper limit. The patient with the highest hTg increase was the only one with markedly elevated TgAB. 

**Figure 1:**
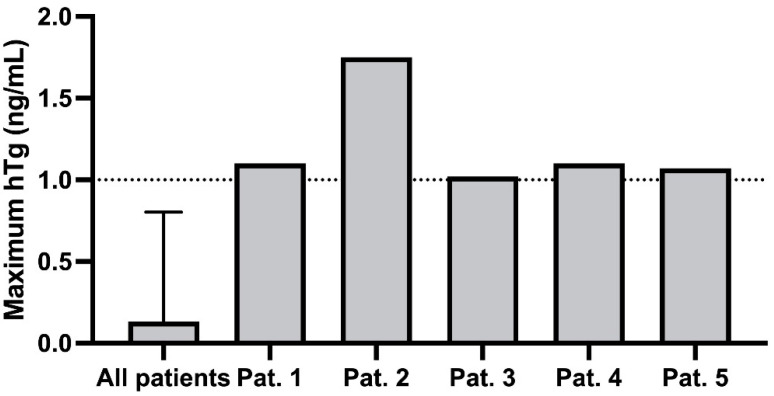
Maximum hTg [ng/mL] of 5 patients with increased hTg ≥ 1 ng/mL.

**Figure 2:**
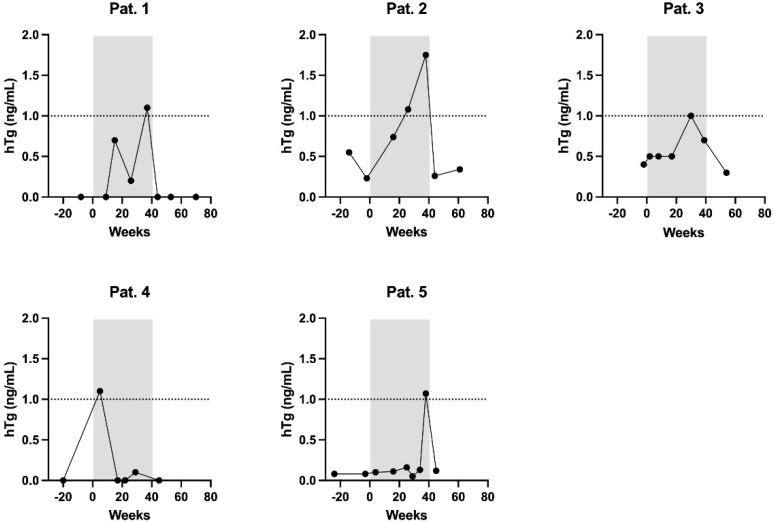
The evolution of the hTG over time of 5 patients with increased hTg ≥ 1 ng/mL.

In total, five patients showed at least one hTg increase to ≥1.0 ng/mL during pregnancy, among these the patient with the advanced tumor stage described above (Figure 1, Table 2). None of these increased levels appeared before the 9th gestational week (Figure 2). Another three patients showed an increase to ≥0.5 ng/mL, at the earliest in the 8th gestational week. Corresponding neck ultrasounds did not show any pathological finding. After delivery, hTg in all of these patients decreased to non-detectable levels (≤0.3 ng/mL), which was why no stimulated hTg determination was carried out. As the specificity of the test kit is high enough to detect significant hTg increases during pregnancy, Friedman test and Holm–Bonferroni method were used. Medians before, during and after pregnancy did not show a significant difference (Friedman´s Chi2 *p* = 0.71, p Holm–Bonferroni = 1.00 for comparison before vs. during, before vs. after and during vs. after pregnancy). For the maximum values, Friedman´s Chi2 as well as p Holm–Bonferroni were significant (*p* << 0.05) comparing hTg before vs. during and hTg during vs. after pregnancy. There was no significant difference comparing hTg before vs. after pregnancy (*p* > 0.05). To the best of our knowledge, eight patients were pregnant more than once. Two of those (one with two and one with three pregnancies) showed hTg levels of 0.5 ng/mL or higher during each pregnancy. In the other six patients (five with two and one with three pregnancies), hTg was detectable at no time. The hTg recovery rate was always within the reference range, thus the results of the hTg measurements can be considered valid. In the further follow-up, none of the patients of the entire collective presented with evidence of recurrence.

## 4. Discussion

Several studies in the current literature investigate the influence of pregnancy on the follow-up care of DTC. So far, mainly the relation between pregnancy and the rate of sonographically detectable recurrence of tumor has been discussed [15]. Murray et al. described a case of a woman with known pulmonary metastasis and a reversible doubling of hTg during pregnancy [16]. However, none of these studies examine the course of hTg in women without morphological and biochemical evidence of recurrent tumor or metastasis. Pomorski et al. did not find a recurrence in 23 patients before, during and after pregnancy regardless of the therapeutic regimen [17]. Rosvoll et al. investigated the follow-up of 38 patients and did not find any recurrence during or after pregnancy [18]. Hill et al. compared 70 patients who became pregnant after DTC had been diagnosed with 109 patients without pregnancy after DTC. The authors did not see a significant difference concerning recurrence rate [19]. Leboeuf et al. retrospectively evaluated 36 patients with DTC and compared the hTg level prior to and after pregnancy. The authors addressed possible laboratory-related effects of pregnancy on follow-up and assumed that pregnancy stimulates the expansion of thyroid cells. In eight out of 36 patients, a 20 % increase in the postpartal hTg was found; however, without statistical significance or detection of a recurrence [20].

Altogether, in our study, five out of 47 women presented with a significant hTg increase causing detectable blood levels during pregnancy, albeit the reason therefore remains unclear. A possible reason might be a placental transfer of fetal hTg, which could be either limited to a minority of individuals, or the transferred amount of hTg in the remaining patients is extremely low and therefore undetectable by the routine follow-up examinations. A placental penetration of hTg has not yet been described in the literature, but is conceivable in cases comparable to the well-known Rh disease [21] with disturbed placental barrier caused by microscopic placental fissures.

Although the terminal differentiation of the human thyroid tissue during fetal development, characterized by follicle formation and onset of thyroid hormone synthesis, is completed in the 11th gestational week, expression of hTg mRNA in fetal thyroid tissue was detected as early as in the 7th gestational week [22]. Furthermore, it is known that even large molecules may pass the placental barrier, e.g., fetal cell-free DNA, arising from apoptosis of fetal hematopoietic cells, that is transferred through the placenta and allows non-invasive prenatal testing [23,24]. As significant hTg levels in pregnant women after complete thyroid ablation were not detected before the 8th gestational week, it seems possible that hTg being measured is fetal hTg that passed through the placental barrier. This hypothesis is supported by the fact that hTg always dropped to non-detectable levels after delivery. Further, in all patients, follow-up neck ultrasounds and hTg determinations did not give any evidence of recurrence. Thyroid tumor progression as a reason of the transitory hTg increase during pregnancy is unlikely. Although there was one patient with a high-risk tumor presenting with an initial hTg level before pregnancy of 0.5 ng/mL, it dropped to 0.3 ng/mL after delivery and the further follow-up revealed no increasing trend. In the latest examination, her hTg level was still 0.6 ng/mL.

The observed increase in TSH levels is common during pregnancy due to the higher demand of thyroid hormones, especially if the dose of the replacement therapy is not adequately adjusted. However, tumor recurrence should not be caused by a short-term TSH increase, and the decrease in hTg concentration afterwards makes this implausible. The decline of a formerly elevated TgAB level during pregnancy in one patient can be attributed to the physiological immune suppression and is thus also not related to a tumor reaction.

A further potential reason for the hTg increase worth discussing could be the corresponding decrease in TgAB over time. Human thyroglobulin antibodies are known to influence the determination of hTg. In the case of the patient with the highest hTg increase up to 1.75 ng/mL, a simultaneous decrease in TgAB during pregnancy was detected (2247 to 1022 U/mL). Human Tg-recovery was not disturbed during the complete period of observation. Despite a normal hTg-recovery, it is possible that the decrease in TgAB caused an increase in the measured hTg [14].

Moreover, it is conceivable that the physiological increase in human choriongonadotropin (hCG) and estrogen caused a stimulation of the secretion of hTg bymicroscopic remnants of thyroid or tumor cells. Estrogen increases the expression of thyroxine binding hormone and, indirectly, the secretion of hTg [25]. There are contradicting statements concerning the development of hCG during pregnancy. Cacciatore B. et al. observed an increasing concentration as soon as five days after conception with a peak level during the 10th to 12th week of gestation (about 230,000 IU/L). Concentration hereafter decreases to 5000–65,000 IU/L towards the end of pregnancy [26]. Kimura et al. stated that hCG shows an intrinsic thyroid stimulating effect comparable to TSH [27]. Therefore, there is a significant correlation between decreasing TSH and increasing hCG level, particularly in the first trimester of pregnancy, caused by a TSH-similar effect of the hCG [27]. Glinoer et al. and Kennedy et al. also found a significant positive correlation between an increasing hCG-level and decreasing TSH in early pregnancy [28,29]. Glinoer et al. attributed the stimulation of the thyroid cells to an increased concentration of the Beta-subunit of hCG in blood serum [28]. However, as there was no evidence of tumor recurrence during the long-term follow-up, either in the imaging or in the laboratory, a proliferation of thyrocytes should not be assumed to be the cause of the temporary rise in hTg in our collective. Limitations of the study are its retrospective nature and the small sample of patients. TgAB positivity and TSH fluctuations in few patients may have affected hTg measurements, thus conclusions drawn must be interpreted with caution.

## 5. Conclusion

In women with thyroid ablative therapy after DTC, a temporary elevation in the hTg level during pregnancy may occur. The reason therefore remains unclear and requires further investigation.

## Figures and Tables

**Table 1:** Patient characteristics.

Variable	
Age at time of diagnosis (years)	24 (14–37)
Age at time of conception (years)	31 (25–41)
Time to conception after last RIT (months)	50 (12–190)
Time to conception after last negative stimulated hTg (months)	21 (6–142)
Histological tumor type	
Papillary carcinoma	45 (96)
Follicular carcinoma	2 (4)
Risk stratification according to ATA guidelines	
Low Risk	11 (23)
High Risk	35 (75)
Unknown	1 (2)

Data presented as median with range or *n* (%).

**Table 2:** Development of hTg, TgAB, hTg-recovery, and TSH of 5 patients with increased hTg > 1 ng/mL.

Patient	hTg [ng/mL]	Tg-AB [U/mL]	hTg Recovery [%]	TSH [mIU/L	Week of Pregnancy
1	0	2	98	17.44	9
	0.7	29	98	4.25	15
	0.2	11	102	1.66	26
	1.1	30	127	1.29	37
2	0.74	2247	89	0.20	16
	1.08	1670	96	0.05	26
	1.75	1022	96	0.03	38
3	0.5	-	104	1.41	8
	0.5	41	96	12.44	17
	1	-	106	3.09	30
	0.7	-	103	1.60	39
4	1.1	16	94	0.04	9
	0	33	97	0.05	17
	0	44	136	0.43	22
	0.1	45	100	1.93	29
5	0.1	5	106	0.31	4
	0.11	7	100	0.42	16
	0.16	15	98	0.24	25
	0.05	5	103	0.11	29
	0.13	12	102	0.12	33
	1.07	8	98	1.68	38

## Data Availability

Not applicable.
